# CYP2C19 genotype-guided escalation to ticagrelor vs. clopidogrel in secondary stroke prevention: a retrospective cohort study

**DOI:** 10.3389/fphar.2026.1747121

**Published:** 2026-02-06

**Authors:** Sun Haidong, Deng Min, Yu Hong, Yang Jing

**Affiliations:** 1 Department of Neurology, Wuhan Hospital of Traditional Chinese Medicine, Wuhan, China; 2 Department of Encephalopathy, Wuhan Hospital of Traditional Chinese Medicine, Wuhan, China; 3 Department of Clinical Laboratory, Wuhan Hospital of Traditional Chinese Medicine, Wuhan, China

**Keywords:** clopidogrel resistance, CYP2C19, genotype-guided therapy, ischemic stroke, secondary prevention, ticagrelor

## Abstract

**Objective:**

To evaluate the effectiveness and safety of an antiplatelet therapy strategy guided by CYP2C19 genotyping in the secondary prevention of ischemic stroke in a real-world setting.

**Methods:**

This single-center retrospective cohort study enrolled 623 ischemic stroke patients. Based on their CYP2C19 genotype (extensive metabolizer [EM], intermediate metabolizer [IM], poor metabolizer [PM]) and the actual P2Y12 receptor antagonist treatment received (clopidogrel or ticagrelor), patients were categorized into natural cohorts. To minimize confounding, we applied propensity score matching, yielding a final analysis cohort of 514 patients. The primary outcome was the incidence of major adverse cardiovascular events (MACE), including stroke recurrence, myocardial infarction, and cardiovascular death, within 12 months. The secondary outcome was bleeding events.

**Results:**

With respect to platelet reactivity, the proportions of high platelet reactivity in PM and IM patients (61.25%, 34.07%) were significantly higher than in EM patients (12.50%) (all *P* < 0.01). Regarding clinical efficacy, among PM and IM patients, the incidence of MACE was significantly lower in the ticagrelor group than in the clopidogrel group (10.00% vs. 30.00%, *P* = 0.025; 11.50% vs. 22.12%, *P* = 0.033, respectively). Among EM patients, there was no significant difference in MACE incidence between the two groups (5.77% vs. 6.73%, *P* = 0.928). After adjustment using Cox regression analysis, ticagrelor therapy emerged as an independent factor associated with a reduced risk of MACE in both PM (HR = 0.32, 95% CI: 0.11–0.89, *P* = 0.029) and IM (HR = 0.52, 95% CI: 0.28–0.98, *P* = 0.043) patients. Furthermore, there were no statistically significant differences in bleeding event rates between the two treatment strategies within any metabolic phenotype (all *P* > 0.05).

**Conclusion:**

Antiplatelet therapy guided by CYP2C19 genotyping is an effective strategy for optimizing the secondary prevention of ischemic stroke. For IM and PM patients, switching from clopidogrel to ticagrelor significantly reduces the risk of recurrent ischemic events without increasing bleeding risk. In contrast, for EM patients, clopidogrel remained an effective and safe option.

## 1 Introduction

Ischemic stroke is a cerebrovascular disease with high incidence, high disability and significant mortality (Chi et al., 2025). Antiplatelet therapy is the core and foundation of secondary prevention of ischemic stroke, and the risk of recurrence can be significantly reduced (Li and Zhao, 2024). Clopidogrel, which is widely used in clinical practice worldwide, irreversibly antagonizes the P2Y12 receptor on the platelet membrane, thereby inhibiting adenosine diphosphate (ADP)-induced platelet aggregation (Xu et al., 2025). However, some patients have insufficient inhibition of platelet aggregation during clopidogrel therapy, i.e. clopidogrel resistance, resulting in an increased risk of recurrence of ischemic events. Pharmacogenomics is a discipline that studies the influence of individual genetic differences on drug response. By detecting gene polymorphisms related to drug metabolism and action, it can provide basis for clinical individualized medication. CYP2C19 gene polymorphism in cytochrome P450 enzyme system is closely related to clopidogrel metabolism and efficacy (Zheng et al., 2019). CYP2C19 gene has multiple alleles, among which *2 and *3 are functional deletion alleles. Carrying these alleles results in absent CYP2C19 enzyme activity. This impairs the metabolism of clopidogrel, leading to reduced production of its active metabolite. Consequently, antiplatelet efficacy is diminished, and the incidence of clopidogrel resistance increases (Botton et al., 2021). Therefore, we conducted this retrospective cohort study to evaluate the effectiveness and safety of a treatment strategy with different P2Y12 receptor antagonists (clopidogrel or ticagrelor) based on CYP2C19 genotype (EM, IM, PM) in the real-world secondary prevention of ischemic stroke.

## 2 Methods

### 2.1 Studay design

This study was a single-center, retrospective cohort study. The study protocol was reported in accordance with the Statement Strengthening the Reporting of Observational Studies in Epidemiology (STROBE). The cohort was naturally formed based on the antiplatelet regimen guided by CYP2C19 genotyping actually received by patients after admission. The core hypotheses were: For CYP2C19 intermediate (IM) and poor metabolizers (PM), switching from clopidogrel to ticagrelor reduces the risk of major adverse cardiovascular events (MACE) without increasing bleeding risk; for patients with extensive metabolizer (EM), there was no significant difference in efficacy and safety between continued clopidogrel and switching to ticagrelor.

### 2.2 Study participants and cohort construction

**FIGURE 1:**
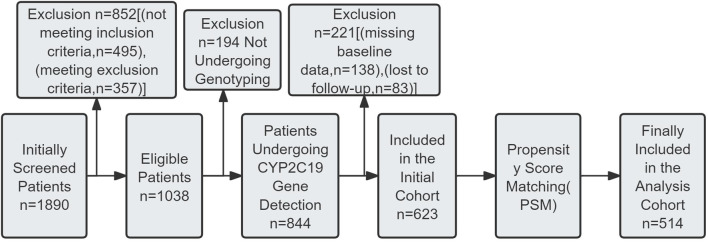
Patient inclusion and exclusion flowchart.

Retrospective collection of medical records of ischemic stroke patients hospitalized in the encephalopathy department of our hospital from January 2019 to December 2023. Inclusion and exclusion flow of study objects is shown in Figure 1. Inclusion criteria: ① Meeting the diagnostic criteria for ischemic stroke in the primary diagnosis and treatment guidelines for ischemic stroke (Chinese Medical Association et al., 2021), and confirmed by brain imaging examination; ② Age 18–65 years old; ③ Admission within 72 h of onset; ④ Mild stroke with NIHSS score ≤5 points; ⑤ No previous use of clopidogrel or other antiplatelet drugs, or discontinuation of drugs for at least 1 week. Exclusion criteria: ① severe liver and kidney dysfunction, hematological diseases, malignant tumors, autoimmune diseases; ② recent history of severe trauma, surgery or hemorrhagic diseases; pregnancy or lactation; ③ allergy to clopidogrel or aspirin. According to the above criteria, 623 patients were included in the initial cohort. To minimize confounding bias in the comparison between treatment strategies, the final analysis cohort was constructed using propensity score matching (PSM) from this initial cohort. The details of the matching procedure are described in Section 2.8. After matching, 514 patients constituted the final cohort for analysis.

### 2.3 Clinical decision pathway and treatment regimens

**FIGURE 2:**
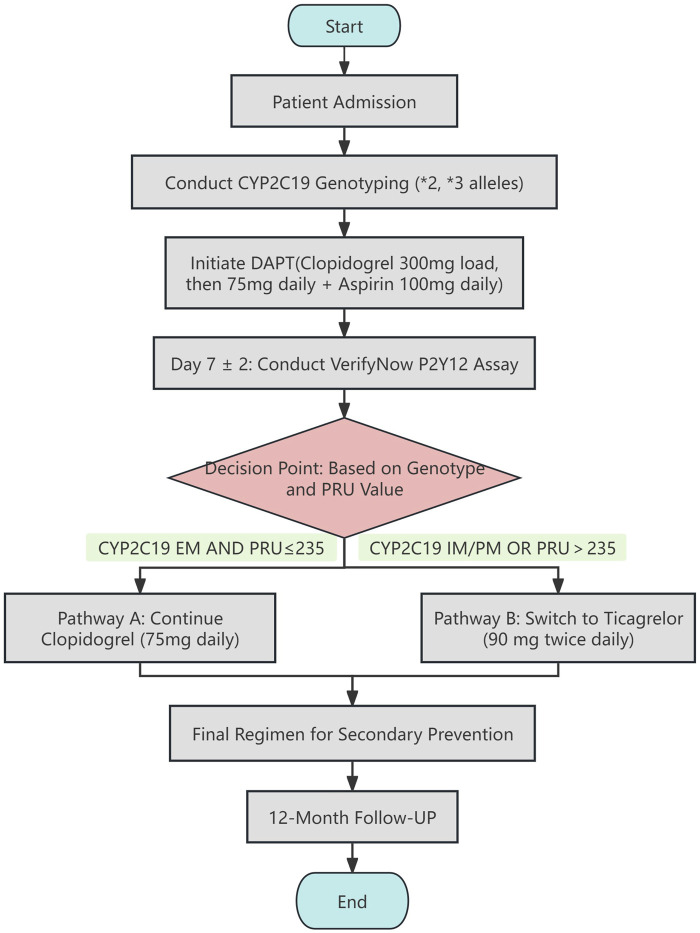
CYP2C19 genotype- and platelet reactivity-guided P2Y12 inhibitor regimen adjustment pathway.

Based on the standardized secondary prevention pathway for ischemic stroke patients established in our center, all enrolled patients received antiplatelet therapy following a sequential decision-making process guided by CYP2C19 genotype and platelet function test results. The specific workflow is as follows (also illustrated in Figure 2):

1. Initial Treatment and Genotyping (At admission, Day 0): At admission, peripheral blood was drawn for immediate CYP2C19 genotyping, which included the *2 (rs4244285) and *3 (rs4986893) alleles. Concurrently, all patients were initiated on guideline-recommended dual antiplatelet therapy, consisting of a loading dose of clopidogrel 300 mg followed by 75 mg daily, plus aspirin 100 mg daily.
2. Platelet Reactivity Assessment (Treatment day 7 ± 2 days): After approximately 7 days of clopidogrel therapy, platelet reactivity was assessed in all patients using the VerifyNow P2Y12 assay, with results expressed in P2Y12 reaction units (PRU). High platelet reactivity (HPR), indicative of clopidogrel hyporesponsiveness, was defined using the established cutoff of PRU >235.
3. Treatment Adjustment Decision (Upon receiving test results, typically between Days 7–10): The attending physician decided on continuing or switching the P2Y12 inhibitor based on the patient’s CYP2C19 phenotype (EM, IM, PM) and HPR status, according to a predefined protocol as follows:

Pathway A (Continue Clopidogrel): Patients who were CYP2C19 EM and showed non-HPR (PRU ≤235) on VerifyNow testing continued the original clopidogrel regimen (75 mg daily).

Pathway B (Switch to Ticagrelor): The P2Y12 inhibitor was switched from clopidogrel to ticagrelor (90 mg twice daily) for patients meeting any of the following criteria:

- CYP2C19 IM or PM phenotype;
- Any metabolic phenotype but with VerifyNow testing showing HPR (PRU >235).

4. Treatment Regimen Finalization and Follow-up: The treatment decision derived from the above test results served as the definitive long-term regimen for secondary prevention. For this retrospective analysis, we adopted an “as-treated” analysis principle. Specifically, patients were categorized into the treatment group (clopidogrel or ticagrelor) corresponding to their final regimen determined after the above decision pathway. Consequently, the final compared groups within each metabolizer phenotype (e.g., clopidogrel vs. ticagrelor in IM patients) reflect the actual treatment received, capturing both adherence to and deviations from the intended genotype-guided protocol in real-world practice. Outcomes were then assessed based on this assignment over a 12-month follow-up period for efficacy and safety. All patients received concomitant aspirin 100 mg daily for 21 days. The total intended duration of antiplatelet therapy was 12 months.

### 2.4 Data collection and baseline characteristics

Baseline data were systematically extracted from the hospital’s electronic medical record system by two independent researchers using a standardized case report form. The extracted information included demographics (age, sex), medical history (hypertension, diabetes, hyperlipidemia, current smoking), clinical severity at admission as assessed by the National Institutes of Health Stroke Scale (NIHSS) score, and laboratory values (low-density lipoprotein cholesterol, LDL-C). Any discrepancies in data extraction were resolved by consensus or adjudication by a third investigator. These characteristics were compared between treatment groups within each CYP2C19 metabolizer phenotype after propensity score matching to ensure balance, as presented in Table 2 of the Results section.

### 2.5 CYP2C19 genotyping and platelet function testing

Peripheral blood was routinely collected at admission for CYP2C19 genotyping. Genomic DNA was extracted and the CYP2C19*2 (rs4244285) and CYP2C19*3 (rs4986893) loci were genotyped using a commercially available real-time fluorescent PCR kit (from Sansure Biotech, Changsha, China) strictly according to the manufacturer’s instructions. Quality control measures included the use of positive and negative controls in each detection batch. Metabolizer interpretation was as follows: patients were classified into CYP2C19 extensive metabolizer (EM, *1/*1), intermediate metabolizer (IM, *1/*2, *1/*3), or poor metabolizer (PM, *2/*2, *3/*3, *2/*3).

Platelet reactivity was quantified using the VerifyNow P2Y12 assay. This system measures the platelet response to adenosine diphosphate (ADP) stimulation and directly reports the degree of P2Y12 receptor pathway inhibition in P2Y12 Reaction Units (PRU). Testing was performed in strict accordance with the manufacturer’s protocol. For this study, high platelet reactivity (HPR) was defined as a PRU value >235. This cutoff is an internationally recognized consensus standard, supported by evidence from large clinical studies (Zhang et al., 2022), and serves to indicate insufficient platelet inhibition under clopidogrel therapy.

### 2.6 Outcome measures

The primary outcome was the incidence of Major Adverse Cardiovascular Events (MACE) within 12 months. MACE was a composite endpoint, including ischemic stroke recurrence, myocardial infarction, and cardiovascular death. The secondary outcome was the incidence of any bleeding event within 12 months, defined according to the International Society on Thrombosis and Haemostasis (ISTH) criteria for clinically relevant bleeding.

Ascertainment of Outcomes: All potential outcome events were captured through a dual approach: ① a systematic review of the hospital’s electronic medical record system for relevant clinical notes, discharge summaries, and laboratory reports; and ② structured telephone interviews conducted at 3, 6, and 12 months using a standardized questionnaire to inquire about interim hospitalizations, new symptoms, and medications. To ensure accuracy, any event reported during telephone follow-up was cross-referenced with available medical records. Final classification of all suspected outcome events was performed by an independent clinical endpoint adjudication committee, blinded to the patients’ CYP2C19 genotype.

### 2.7 Bias control

In order to reduce information bias in retrospective studies, the following measures were taken: ① Data were extracted from electronic medical records system by double independent back-to-back method (Including baseline data, gene test results, platelet function test results and outcome events), cross-check after extraction, and adjudicate by the third investigator in case of disagreement; ② Conduct unified training for investigators involved in data collection and outcome interpretation, and use standardized case report forms; ③ For outcome events obtained by telephone follow-up, strive to verify by referring to readmission medical records.

### 2.8 Statistical method

All statistical analyses were performed using SPSS (version 26.0; IBM Corp.) and R (version 4.3.0; R Foundation for Statistical Computing). Given the observational nature of this study, we used PSM to mitigate potential confounding. Specifically, propensity scores were estimated separately for the EM, IM, and PM groups using a multivariable logistic regression model, with treatment assignment (ticagrelor vs. clopidogrel) as the outcome and the following clinically relevant covariates as predictors: age, sex, history of hypertension, history of diabetes, current smoking, and baseline NIHSS score. Matching was then performed 1:1 using nearest-neighbor matching without replacement and a caliper width set at 0.02 of the propensity score’s logit standard deviation.

Covariate balance between treatment groups before and after matching within each metabolizer group was assessed using standardized mean differences (SMDs), with an SMD <0.10 generally considered indicative of adequate balance. The detailed results of this balance assessment for each phenotype group are provided in Supplementary Table S1. As shown in Supplementary Table S1 and Table 1, propensity score matching substantially reduced the SMDs for most covariates, thereby improving comparability between groups. Although the majority of post-matching SMDs were below or close to the 0.10 threshold, some mild residual imbalances persisted in specific subgroups, such as age in the EM and PM groups. These residual imbalances were subsequently adjusted for in the multivariate Cox regression analyses. Detailed results of the covariate balance improvement achieved through matching are provided in Supplementary Table S1 and summarized in the Results section.

**TABLE 1:** Baseline characteristics of the study cohort before and after propensity score matching.

Baseline characteristic	Before PSM (n = 623)		SMD (before)	After PSM (n = 514)		SMD (after)
	Clop. (n = 308)	Tica. (n = 315)		Clop. (n = 257)	Tica. (n = 257)	
Age (years)	57.44 ± 10.81	60.12 ± 9.68	0.261	58.02 ± 10.31	59.26 ± 9.49	0.125
Male, n (%)	179 (58.12%)	164 (52.06%)	0.122	137 (53.31%)	133 (51.75%)	0.031
Hypertension, n (%)	187 (60.71%)	207 (65.71%)	0.104	162 (63.04%)	167 (64.98%)	0.040
Diabetes, n (%)	121 (39.29%)	144 (45.71%)	0.130	111 (43.19%)	119 (46.30%)	0.063
Smoking, n (%)	140 (45.45%)	127 (40.32%)	0.104	112 (43.58%)	105 (40.86%)	0.055
NIHSS score (x ± s)	3.42 ± 1.52	3.04 ± 1.84	0.225	3.35 ± 1.55	3.18 ± 1.66	0.106
LDL-C, mmol/L	3.70 ± 1.49	3.99 ± 1.62	0.186	3.80 ± 1.47	3.94 ± 1.43	0.097

Data are presented as mean ± standard deviation or number (percentage). Clop., clopidogrel group; Tica., ticagrelor group; NIHSS, national institutes of health stroke scale; LDL-C, low-density lipoprotein cholesterol.

After PSM, continuous variables were compared using the independent samples *t*-test or Mann-Whitney U test, as appropriate, and are presented as mean ± standard deviation or median (interquartile range). Categorical variables were compared using the Chi-square test or Fisher’s exact test and are presented as frequencies (percentages). The primary outcome of MACE was analyzed using multivariable Cox proportional hazards regression models, stratified by metabolizer group. To account for any residual confounding despite matching, these Cox models were additionally adjusted for the same set of covariates used in the propensity score estimation. Results are expressed as hazard ratios (HR) with 95% confidence intervals (CI). The proportional hazards assumption was verified using Schoenfeld residual plots. A two-sided P value <0.05 was considered statistically significant.

### 2.9 Ethics

This study was approved by the Ethics Committee of Wuhan Hospital of Traditional Chinese Medicine (ethical batch number: Wu Zhongyi Lun QT2025-086-01(C)). All data were processed anonymously, which complied with the principles of Declaration of Helsinki.

## 3 Results

### 3.1 Patient baseline characteristics after PSM

PSM substantially improved the balance of predefined covariates across the cohort, as detailed in Supplementary Table S1. This yielded a final matched cohort of 514 patients for analysis. The baseline characteristics of this well-matched cohort, stratified by CYP2C19 metabolizer phenotype and treatment group, are presented in Table 2.

**TABLE 2:** Baseline characteristics of the propensity score-matched cohort, stratified by CYP2C19 metabolizer phenotype and treatment group.

Baseline characteristics	EM type (n = 208)			IM type (n = 226)			PM type (n = 80)		
	Clop. (n = 104)	Tica. (n = 104)	SMD	Clop. (n = 113)	Tica. (n = 113)	SMD	Clop. (n = 40)	Tica. (n = 40)	SMD
Age (year)	58.15 ± 10.14	60.02 ± 9.31	0.191	57.63 ± 11.03	58.25 ± 10.66	0.057	58.78 ± 10.65	60.15 ± 9.64	0.134
Male, n (%)	54 (51.92%)	56 (53.85%)	0.039	61 (53.98%)	57 (50.44%)	0.071	22 (55.00%)	20 (50.00%)	0.100
Hypertension, n (%)	67 (64.42%)	71 (68.27%)	0.081	69 (61.06%)	71 (62.83%)	0.036	26 (65.00%)	25 (62.50%)	0.052
Diabetes, n (%)	49 (47.12%)	54 (51.92%)	0.096	45 (39.82%)	50 (44.25%)	0.090	17 (42.50%)	15 (37.50%)	0.102
Smoking, n (%)	41 (39.42%)	39 (37.50%)	0.040	51 (45.13%)	48 (42.48%)	0.054	20 (50.00%)	18 (45.00%)	0.100
NIHSS score (x ± s)	3.32 ± 1.43	3.17 ± 1.64	0.097	3.41 ± 1.31	3.24 ± 1.64	0.114	3.25 ± 1.52	3.05 ± 1.72	0.122
LDL-C, mmol/L	3.71 ± 1.41	3.84 ± 1.47	0.090	3.91 ± 1.51	4.05 ± 1.44	0.095	3.71 ± 1.38	3.90 ± 1.52	0.130

Data are presented as mean ± standard deviation or number. NIHSS, national institutes of health stroke scale; LDL-C, low-density lipoprotein cholesterol. There were no significant differences in all baseline characteristics between the clopidogrel and ticagrelor groups within any metabolizer group (all P > 0.05).

### 3.2 Platelet reactivity comparison

CYP2C19 PM type patients HPR proportion was 61.25% (49/80), significantly higher than IM type 34.07% (77/226) and EM type 12.50% (26/208), the difference was statistically significant (P < 0.05). IM type patients HPR proportion was also significantly higher than EM type (P < 0.05), as shown in Table 3.

**TABLE 3:** Comparison of HPR in patients with different CYP2C19 metabolizers in matched cohorts.

Metabolic type	number(*n*)	HPR number(*n*)	HPR rate (%)	Comparison group	*χ* ^*2*^	*P*
EM type	208	26	12.50	EM vs. IM	31.07	<0.01
IM type	226	77	34.07	EM vs. PM	72.32	<0.01
PM type	80	49	61.25	IM vs. PM	17.29	<0.01

Data in this table are based on propensity score-matched cohorts, HPR: high platelet reactivity.

### 3.3 Comparison of incidence of MACE in patients with different treatment regimens

At 12 months follow-up, the incidence of MACE was 30.00% (12/40) in CYP2C19PM patients who continued clopidogrel treatment, significantly higher than 10.00% (4/40) in patients who were escalated to ticagrelor, the difference was statistically significant (P < 0.05). Among CYP2C19 IM patients, the incidence of MACE was 22.12% (25/113) in patients who continued clopidogrel treatment, significantly higher than 11.50% (13/113) in patients who were titrated to ticagrelor, the difference was statistically significant (P < 0.05). Among CYP2C19EM patients, the incidence of MACE was 6.73% (7/104) in patients who continued clopidogrel treatment, compared with 5.77% (6/104) in patients who were titrated to ticagrelor, and the difference was not statistically significant (P = 0.928), as shown in Table 4.

**TABLE 4:** Comparison of incidence of MACE among patients with different treatment regimens in matched cohort (%).

Metabolic type	Therapeutic schedule	Number	Number of MACE (*n*)	Incidence of MACE (%)	*χ* ^*2*^	*P*
EM type	Clop.	104	7	6.73	-	-
	Tica.	104	6	5.77	0.01	0.928
IM type	Clop.	113	25	22.12	-	-
	Tica.	113	13	11.50	4.55	0.033
PM type	Clop.	40	12	30.00	-	-
	Tica.	40	4	10.00	5.00	0.025

Data in this table are based on propensity score-matched cohorts. MACE: major adverse cardiovascular event.

### 3.4 Comparison of bleeding events among patients treated with different regimens

During the follow-up period, there was no statistically significant difference in the incidence of bleeding events between patients with different CYP2C19 metabolisms and patients with different treatment regimens (all P > 0.05), as shown in Table 5.

**TABLE 5:** Comparison of incidence of bleeding events among patients with different treatment regimens in matched cohort (%).

Metabolic type	Therapeutic schedule	number(*n*)	Total number of bleeds(*n*)	Total bleeding rate(*%*)	*χ* ^*2*^	*P*
EM type	Clop.	104	7	6.73	-	-
	Tica.	104	8	7.69	0.07	0.791
IM type	Clop.	113	13	11.50	-	-
	Tica.	113	10	8.85	0.43	0.512
PM type	Clop.	40	5	12.50	-	-
	Tica.	40	4	10.00	0.13	0.718

Data in this table are based on propensity score-matched cohorts.

### 3.5 Multivariate cox regression analysis

To confirm the independent effect of treatment regimen on the risk of MACE, we further performed a multivariate Cox proportional hazards regression analysis, which, after adjusting for age, sex, history of hypertension, diabetes, and smoking history (Table 6), showed that ticagrelor treatment significantly reduced the risk of MACE compared with clopidogrel treatment in CYP2C19 PM patients (HR = 0.32, 95%CI:0.11–0.89, P = 0.029). Ticagrelor treatment also significantly reduced the risk of MACE in CYP2C19 IM patients (HR = 0.52, 95%CI:0.28–0.98, P = 0.043). In CYP2C19EM patients, there was no significant difference in risk between the two treatment regimens (HR = 1.05, 95%CI:0.36–3.02, P = 0.931). This result further confirms the independent value of adjusting treatment strategies based on genotype.

**TABLE 6:** Multivariate Cox regression analysis of MACE risk in patients with different CYP2C19 metabolizers.

Metabolic type	Variable	HR	95%CI	P值
EM type	Tica. (vs. Clop.)	1.05	0.36–3.02	0.931
	Age (for each additional year)	1.02	0.96–1.09	0.512
	Gender (M vs. F)	1.21	0.41–3.59	0.731
	Hypertension (yes vs. no)	1.35	0.45–4.08	0.592
	Diabetes (yes vs. no)	1.58	0.52–4.76	0.416
	Smoking (yes vs. no)	1.12	0.38–3.31	0.836
IM type	Tica. (vs. Clop.)	0.52	0.28–0.98	0.043
	Age (for each additional year)	1.03	0.99–1.07	0.105
	Gender (M vs. F)	1.18	0.64–2.18	0.601
	Hypertension (yes vs. no)	1.29	0.69–2.41	0.426
	Diabetes (yes vs. no)	1.45	0.78–2.70	0.241
	Smoking (yes vs. no)	1.09	0.59–2.01	0.782
PM type	Tica. (vs. Clop.)	0.32	0.11–0.89	0.029
	Age (for each additional year)	1.04	0.97–1.12	0.279
	Gender (M vs. F)	1.41	0.55–3.62	0.473
	Hypertension (yes vs. no)	1.62	0.63–4.17	0.318
	Diabetes (yes vs. no)	1.78	0.69–4.55	0.230
	Smoking (yes vs. no)	0.85	0.33–2.17	0.730

MACE, major adverse cardiovascular event;HR, hazard ratio; CI, confidence interval. Analysis was based on propensity score-matched cohort and adjusted for covariates listed in table.

## 4 Discussion

This real-world retrospective cohort study provides evidence that a CYP2C19 genotype-guided strategy for selecting P2Y12 receptor antagonists may optimize the secondary prevention of ischemic stroke. Our findings suggest that for patients identified as CYP2C19 intermediate or poor metabolizers, escalating therapy from clopidogrel to ticagrelor was associated with a lower risk of recurrent MACE, without a significant increase in bleeding events. In contrast, for extensive metabolizers, clopidogrel appeared to maintain comparable effectiveness and safety, highlighting the potential value of genotyping in personalizing therapy and avoiding unnecessary treatment escalation.

Our findings robustly reinforce the pivotal role of CYP2C19 polymorphisms in determining clopidogrel response. The observed gradient in HPR rates—from 12.50% in EMs to 34.07% in IMs and 61.25% in PMs—neatly illustrates the gene-dose effect and provides a compelling pharmacodynamic explanation for the ensuing clinical outcomes. This pattern, well-documented in patients undergoing percutaneous coronary intervention (Coons et al., 2022; Kim et al., 2023), is now convincingly replicated in a dedicated ischemic stroke cohort, underscoring the consistency of this pharmacogenetic relationship across different atherosclerotic diseases (e.g., coronary and cerebral arteries). The suboptimal inhibition of platelet aggregation in IM and PM patients directly translates into tangible clinical harm, as evidenced by the significantly higher MACE incidence in these groups when treated with clopidogrel.

A key contribution of our analysis is the validation of a genotype-guided “precision escalation” strategy. The multivariate Cox regression confirmed ticagrelor as a powerful, independent protective factor against MACE, reducing the risk by 68% in PMs and 48% in IMs. This result is strongly aligned with a growing body of evidence from the CHANCE-2 trial and its subsequent analyses, which confirmed the superiority of ticagrelor over clopidogrel in CYP2C19 loss-of-function allele carriers with minor stroke or TIA (Xie et al., 2023), including in specific subgroups (Wu et al., 2025) and over extended follow-up (Xia et al., 2024). A systematic review further consolidates this evidence, highlighting the consistent efficacy and safety profile of ticagrelor in this genetically defined population (Li et al., 2024). Ticagrelor, by virtue of its direct and reversible mechanism of action that bypasses CYP2C19-mediated metabolism, constitutes a reliable therapeutic alternative for clopidogrel-resistant patients (Wang et al., 2022; Smith et al., 2024). Reassuringly, despite its more potent antiplatelet effect, we observed no statistically significant increase in bleeding risk with ticagrelor across all metabolic phenotypes. This safety profile, consistent with findings from the THALES trial (Wang et al., 2023), suggests a favorable net clinical benefit. The reversible binding nature of ticagrelor may contribute to this preserved hemostatic balance (Aytekin et al., 2024).

Our results also present nuances that warrant careful interpretation. First, we noted an intriguing pattern: within the EM patients, the MACE rate in the ticagrelor group (5.77%) was numerically lower than in the clopidogrel group (6.73%), despite the lack of statistical significance. As ticagrelor’s effect is not mediated by CYP2C19, its efficacy would theoretically be similar across metabolizer phenotypes. This trend toward an “additional” benefit of ticagrelor in EMs is most likely attributable to residual confounding or chance. In clinical practice, physicians might have been more inclined to prescribe ticagrelor to EM patients with unmeasured adverse prognostic features (e.g., perceived poor adherence or risk from other genetic variants). While PSM balanced measured covariates, it cannot fully eliminate such channeling bias based on clinical judgment. ∼∼Second, Table 2 shows a non-significant trend toward higher baseline NIHSS scores among those switched to ticagrelor in the IM and PM groups, again suggesting a potential clinical inclination to escalate therapy in patients perceived as more severe.∼∼ These subtle patterns do not undermine our core conclusion regarding the overall value of genotype-guided therapy but rather highlight how the complexity of real-world decision-making can leave traces of residual confounding even after rigorous statistical adjustment. They underscore the importance of confirming these findings in a prospective, randomized setting.

From a clinical and health economic perspective, implementing this strategy for the approximately 60% of non-EM patients in our cohort holds significant promise. It enables the targeted use of a more potent, albeit costlier, agent in those who stand to benefit most, while safely conserving healthcare resources by continuing generic clopidogrel in EM patients. An analysis based on the THALES trial already suggests this approach is cost-effective in the Chinese healthcare setting for patients with mild stroke/TIA (Tank et al., 2023).

Several limitations of this study should be considered. First, despite the rigorous application of propensity score matching to balance measured confounders (as shown in Supplementary Table S1), its single-center, retrospective observational design remains susceptible to residual confounding by unmeasured factors (e.g., medication adherence, socioeconomic status) and selection bias. Second, the sample size, particularly for the poor metabolizer (PM) subgroup (n = 40), limited the statistical power for analyzing safety outcomes such as bleeding events and resulted in wide confidence intervals. Third, the follow-up duration was 12 months; longer-term efficacy and safety beyond 1 year warrant further investigation. Fourth, our genotyping was limited to the CYP2C19 *2 and *3 loss-of-function alleles. We did not test for the CYP2C19*17 gain-of-function allele, which is part of the minimum allele set for comprehensive CYP2C19 phenotyping and could influence clopidogrel response and bleeding risk. The absence of *17 data represents a limitation in our genotype-guided classification. Future prospective, genotype-guided randomized trials with larger samples and longer follow-up are needed to confirm our findings.

In conclusion, CYP2C19 genotype-guided antiplatelet therapy represents a pragmatic and effective path toward personalizing secondary stroke prevention. It allows for the precise identification of patients at high risk for clopidogrel treatment failure and safely directs them toward more effective alternatives, thereby optimizing both clinical outcomes and resource allocation.

## Data Availability

The original datasets (including clinical records and genotype data) generated and analyzed during this study are not publicly available due to patient privacy and confidentiality restrictions, as stipulated by the Ethics Committee of Wuhan Hospital of Traditional Chinese Medicine. However, de-identified data supporting the findings of this study are available from the corresponding author (Min Deng) upon reasonable request for academic and non-commercial purposes, subject to approval by the aforementioned Ethics Committee.
