# Supplementary material for: CYP2C19 genotype-guided escalation to ticagrelor vs. clopidogrel in secondary stroke prevention: a retrospective cohort study
Source: Front Pharmacol. 2026 Feb 6;17:1747121. doi: 10.3389/fphar.2026.1747121 (PMC12920421; doi:10.3389/fphar.2026.1747121)
Supplement: Supplementary file 1 [file Table1.docx]

Supplementary Table S1. Covariate Balance Before and After Propensity Score Matching Within Each CYP2C19 Metabolizer Group

| CYP2C19 Phenotype | Covariate | Before PSM | | SMD (Before) | After PSM | | SMD (After) |
| --- | --- | --- | --- | --- | --- | --- | --- |
|  |  | Clop. | Tica. |  | Clop. | Tica. |  |
| EM | Total n | 126 | 114 | - | 104 | 104 | - |
|  | Age, years | 56.80±11.05 | 60.43±9.42 | 0.351 | 58.15±10.14 | 60.02±9.31 | 0.191 |
|  | Male, n (%) | 72 (57.14%) | 70(61.40%) | 0.087 | 54 (51.92%) | 56(53.85%) | 0.039 |
|  | Hypertension, n (%) | 75 (59.52%) | 82(71.93%) | 0.261 | 67 (64.42%) | 71(68.27%) | 0.081 |
|  | Diabetes, n (%) | 52 (41.27%) | 63(55.26%) | 0.280 | 49 (47.12%) | 54(51.92%) | 0.096 |
|  | Smoking, n (%) | 56 (44.44%) | 48(42.11%) | 0.047 | 41 (39.42%) | 39(37.50%) | 0.040 |
|  | NIHSS score | 3.35 ± 1.55 | 3.05 ±1.82 | 0.178 | 3.32 ± 1.43 | 3.17 ± 1.64 | 0.097 |
|  | LDL-C, mmol/L | 3.60 ± 1.53 | 3.92 ±1.59 | 0.204 | 3.71 ± 1.41 | 3.84 ±1.47 | 0.090 |
| IM | Total n | 129 | 139 | - | 113 | 113 | - |
|  | Age, years | 58.00±10.70 | 59.48±9.88 | 0.144 | 57.63±11.03 | 58.25±10.66 | 0.057 |
|  | Male, n (%) | 77 (59.69%) | 69(49.64%) | 0.202 | 61 (53.98%) | 57 (50.44%) | 0.071 |
|  | Hypertension, n (%) | 79 (61.24%) | 91(65.47%) | 0.088 | 69 (61.06%) | 71 (62.83%) | 0.036 |
|  | Diabetes, n (%) | 48 (37.21%) | 61(43.88%) | 0.136 | 45 (39.82%) | 50 (44.25%) | 0.090 |
|  | Smoking, n (%) | 60 (46.51%) | 58(41.73%) | 0.096 | 51 (45.13%) | 48 (42.48%) | 0.054 |
|  | NIHSS score | 3.55 ± 1.48 | 3.05 ± 1.86 | 0.298 | 3.41 ± 1.31 | 3.24 ± 1.64 | 0.114 |
|  | LDL-C, mmol/L | 3.82 ± 1.45 | 4.06 ± 1.61 | 0.154 | 3.91 ± 1.51 | 4.05 ± 1.44 | 0.095 |
| PM | Total n | 53 | 62 | - | 40 | 40 | - |
|  | Age, years | 57.60±11.00 | 60.98±9.85 | 0.323 | 58.78±10.65 | 60.15± 9.64 | 0.134 |
|  | Male, n (%) | 30 (56.60%) | 25(40.32%) | 0.326 | 22 (55.00%) | 20 (50.00%) | 0.100 |
|  | Hypertension, n (%) | 33 (62.26%) | 34(54.84%) | 0.151 | 26 (65.00%) | 25 (62.50%) | 0.052 |
|  | Diabetes, n (%) | 21 (39.62%) | 20(32.26%) | 0.154 | 17 (42.50%) | 15 (37.50%) | 0.102 |
|  | Smoking, n (%) | 24 (45.28%) | 21(33.87%) | 0.231 | 20 (50.00%) | 18 (45.00%) | 0.100 |
|  | NIHSS score | 3.30 ± 1.56 | 3.00 ± 1.86 | 0.178 | 3.25 ± 1.52 | 3.05 ± 1.72 | 0.122 |
|  | LDL-C, mmol/L | 3.65 ± 1.51 | 3.95 ± 1.69 | 0.186 | 3.71 ± 1.38 | 3.90 ± 1.52 | 0.130 |
